# Flexible Piezoelectric Generators by Using the Bending Motion Method of Direct-Grown-PZT Nanoparticles on Carbon Nanotubes

**DOI:** 10.3390/nano7100308

**Published:** 2017-10-07

**Authors:** Jin Kyu Han, Do Hyun Jeon, Sam Yeon Cho, Sin Wook Kang, Jongsun Lim, Sang Don Bu

**Affiliations:** 1 Department of Physics and Research Institute of Physics and Chemistry, Chonbuk National University, Jeonju 54896, Korea; jkhan@jbnu.ac.kr (J.K.H.); dhjeon@jbnu.ac.kr (D.H.J.); syc@jbnu.ac.kr (S.Y.C.); swkang@jbnu.ac.kr (S.W.K.); 2 Thin Film Materials Research Center, Korea Research Institute of Chemical Technology (KRICT), Daejeon 34114, Korea; jslim@krict.re.kr

**Keywords:** piezoelectric nanogenerator, composite material, bending movement

## Abstract

Recently, composite-type nanogenerators (NGs) formed from piezoelectric nanostructures and multi-walled carbon nanotubes (CNTs), have become one of the excellent candidates for future energy harvesting because of their ability to apply the excellent electrical and mechanical properties of CNTs. However, the synthesis of NG devices with a high proportion of piezoelectric materials and a low polymer content, such as of polydimethylsiloxane (PDMS), continues to be problematic. In this work, high-piezoelectric-material-content flexible films produced from Pb(Zr,Ti)O_3_ (PZT)-atomically-interconnected CNTs and polytetrafluoroethylene (PTFE) are presented. Various physical and chemical characterization techniques are employed to examine the morphology and structure of the materials. The direct growth of the piezoelectric material on the CNTs, by stirring the PZT and CNT mixed solution, results in various positive effects, such as a high-quality dispersion in the polymer matrix and addition of flexoelectricity to piezoelectricity, resulting in the enhancement of the output voltage by an external mechanical force. The NGs repeatedly generate an output voltage of 0.15 V. These results present a significant step toward the application of NGs using piezoelectric nanocomposite materials.

## 1. Introduction

Renewable energy harvesting through piezoelectric nanogenerators (PNGs) has become one of the most important requirements to resolve the energy crisis arising from the highly increased energy consumption, a result of burning fossil fuels [1,2,3,4]. Recently, there have been various reports of PNGs composed of one-dimensional perovskite-type nanostructures such as Pb(Zr,Ti)O_3_ (PZT) nanowires [5], Pb(Mg,Nb)O_3_-PbTiO_3_ nanowires [6], (K,Na)NbO_3_ nanorods [7], PbTiO_3_ (PTO) [8], and BaTiO_3_ (BTO) nanotubes [9]; these materials are used owing to their outstanding piezoelectric properties. However, these nanostructures, including oxide materials, are difficult to employ for PNG fabrication because they are quite brittle and rigid. To avoid splitting and cleavage during the fabrication process and to enhance the endurance and flexibility of the PNGs, composite-type PNGs composed of piezoelectric materials and flexible one-dimensional structures, such as carbon nanotubes (CNTs) [10,11,12,13] and viruses [14], have been utilized. Such composites employ a polymer matrix, such as polydimethylsiloxane (PDMS) that improves the flexibility, but lowers the power generation properties by limiting the amount of the piezoelectric material in the sample.

In this study, we fabricate a film by mixing PZT nanoparticles (NPs) (PZTNPs) directly grown on a CNT (PZTNP-CNT) with a polymer binder called polytetrafluoroethylene (PTFE) to increase the amount of the piezoelectric material while simultaneously maintaining the flexibility. Through the use of PTFE, which has the property of fiber type, it is possible to fabricate PNG devices with the maximum amount of piezoelectric materials. As the amount of the piezoelectric substance increases, the piezoelectric substance can be close to each other, thereby helping the strain to be more actively transferred by the external force. The PTFE content is 20%, and so, approximately 40% or more of the PZTNPs are present in the entire sample. Mechanical deformation of our PZTNP-CNT nanogenerator by a bending machine generates a maximum output voltage of 0.19 V. The output voltage is about five times higher than the PNGs using simply mixed composites. Also, the reaction time in the banding motion of PZTNP-CNT was faster than that of simply mixed composites. In order to understand the enhancement of the output power, we investigated the atomic-scale strain distribution of epitaxial PZTNPs grown directly on CNT prepared. The PZTNP-CNT nanogenerators, together with the soft nature of our CNT devices, pave the way for diverse and adaptable applications, such as mobile electronics, health monitoring, mapping, and compensation in wearable electronics in the future. Moreover, our results represent a momentous step towards the application of energy harvesting in flexible electronics, portable devices, and mechanical sensors.

## 2. Results and Discussion

**Figure 1:**
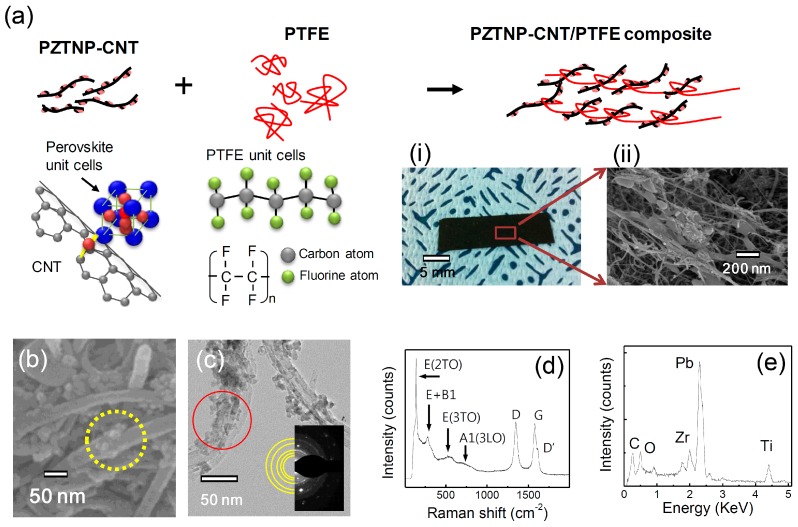
(**a**) Schematic of the fabrication process of a Pb(Zr,Ti)O_3_ (PZT)NP-carbon nanotubes (CNT) film using polytetrafluoroethylene (PTFE), (i) photograph and (ii) field emission scanning electron microscopy (FESEM) of a PZTNP-CNT film with a thickness of approximately 100 μm; and (**b**) FESEM; (**c**) FETEM images; and (**d**) Raman spectrum of PZTNP-CNT; (**e**) X-ray analysis (EDX) spectrum of PZTNP-CNT.

Figure 1a is a schematic of the fabrication process of a PZTNP-CNT film using PTFE. The perovskite-structured PZTNP-grown CNT surface was fabricated by sol-gel synthesis and a filtering method. In the yellow circle in Figure 1b displaying the field emission scanning electron microscopy (FESEM) image, particles of approximately 20 nm to 50 nm in size are observed on the CNTs. Figure 1c presents the field emission transmission electron microscopy (FETEM) images of the PZTNP-CNT film, showing that the PZTNPs are distributed more on the outer surface of the CNTs than on their inside. The PZTNPs nucleated at the carboxylic groups in the defects of CNTs that were introduced by a functionalizing process involving a reaction with HNO_3_ acid solution [15,16].

In the selected area electron diffraction (SAED) patterns in inset images of Figure 1c, the measured lattice spacings of 2.84, 2.35, 2.07, 1.44, and 1.28 Å in the NPs correspond to the perovskite-structured PZT (101), (111), (002), (022), and (310) reflections, respectively, indicating that the NPs are polycrystalline. Figure 1d presents Raman spectra of PZTNP-CNT, which is a sensitive way to identify tetragonal symmetry of the perovskite structure. In the Raman spectrum, 3A1 + B1 + 4E modes of tetragonal PZT are found, indicating the space group P4 mm. Raman peaks at 205, 275, 533, and 724 cm^−1^ are corresponded to E(2TO), E+B1, E(3TO), and A1(3LO) of PZT, respectively. These phonon modes are consistent with the PZT perovskite phase. Therefore, we can confirm that PZT is crystallized in PZTNP-CNT. Moreover, bands at 1330, 1581, and 1616 cm^−1^ are observed, indicating disordered sp^2^ carbon in the CNTs (denoted by the D band), well-ordered graphite structures (denoted by the G band), and end planes of graphene layers (denoted by the D’ band), respectively. This means that the structure of CNT in PZTNP-CNT is stable.

Figure 1e displays the energy dispersive X-ray analysis (EDX) spectrum using a FETEM of PZTNP-CNT as indicated by the solid red circle in Figure 1c, in which C element of the CNT and Pb, Zr, Ti, and O elements of PZT are identified. These observations confirm that the PZTNPs are formed well with a perovskite structure on the CNTs. The PZTNP-CNT films with a thickness of approximately 100 μm are prepared as shown in Figure 1a (i) and (ii) by mixing PZTNP-CNT with PTFE. PTFE is composed of a chain of carbon atoms with two fluorine atoms on each carbon. The chain binds to the PZTNP-CNT powder, forming a 2-dimensional film. The mixture of 80 wt % PZTNP-CNT and 20 wt % PTFE is well mixed, and then pressed by a rolling machine, until a thickness of 180 μm is attained. PZTNP-CNT is well-retained in a film form by PTFE because its flexible nature with a thread-like chain form. This feature allows the formation of a flexible film that does not crack or damage even after repetitive movements such as bending. The film was subsequently attached to a polyimide (PI) substrate coated with Au using carbon paste. An Au electrode was deposited on it, and the output voltage was measured at the time of bending by connecting an oscilloscope to both the electrodes using metal wires.

**Figure 2:**
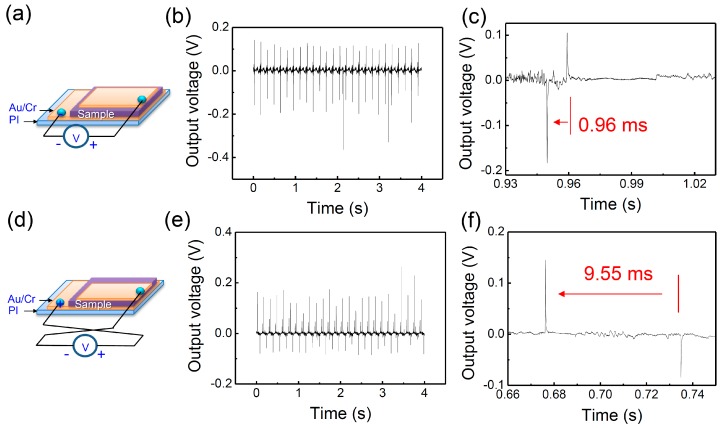
Nanogenerators (NG) properties showing polarity in (**a**–**c**) forward and (**d**–**f**) reverse connection.

First, as shown in the schemes of Figure 2a,d, the polarity in the bending movement is confirmed by the switching of the direction of the electrode. Here, the bending was applied at a strain of approximately 0.68%, and an external resistance of 60 MΩ was connected. The strain applied to the device is calculated using the equation given by

(1) $$\varepsilon =\frac{X}{R}$$

where ε is the strain (dimensionless), *X* is the distance from the neutral plain (m), and *R* is the radius of curvature (m). The strain is inversely proportional to the bending radius and directly proportional to the distance from the neutral mechanical plane. The bending radius is 9.5 mm that is measured at the center between the bending clips, where the bending radius has a maximum value. The neutral mechanical plane was determined from the thickness and elastic modulus of each layer, and was found to be 550 μm.

The output voltages were measured in reverse directions to confirm that the signals were generated from the piezoelectric property of the PZTNP-CNT films. When the connection was switched, the polarity inversion of the output signals was observed, as shown in Figure 2b,e. When using a device with only CNTs, the NG property was found to be nearly negligible, and the device did not show the polarity characteristics as compared with PZTNP-CNT (Supplementary Materials, Figure S2). According to Figure 2b,e, the forward and reverse connection output voltages are 0.15 V and 0.19 V, respectively. Through this switching, we can deduce that the signals are a result of the piezoelectricity of PZTNP-CNT.

The power from the NGs of PZTNP-CNT was calculated from the following equation:

(2) $$P=\frac{1}{T}\int \frac{V_{0}{\left(t\right)}^{2}}{R}dt$$

where *T* is the period of bending movement, *V*_0_(*t*) is the real-time output voltage, and *R* is the external resistance. When the external resistance is 60 MΩ, volume power density exhibited the largest value of 0.2 nW/cm^2^. As the external resistance increased to 6 GΩ, the NG characteristic became smaller and showed an asymmetry tendency, as shown in Figure S1. Table S1 shows the NG characteristics of PZT nanostructures reported previously. Mostly, external force is applied by tapping movement by pressing with Teflon stack, however there are not many reports of NG using bending movements. As shown in Figure 2c,f, the shape of the voltage output signal within a pitch is very repeatable across pitches, where the output voltage is close to sinusoidal (e.g., alternating current). In addition, the time difference between the signals during bending and releasing is 0.96 ms for the forward connection and 9.55 ms for the reverse connection. In the forward connection, the time difference approximately 10 times faster than that of the reverse connection because the film and bottom electrode are connected by a carbon paste used as an adhesive, while the film and top electrode are directly connected.

**Figure 3:**
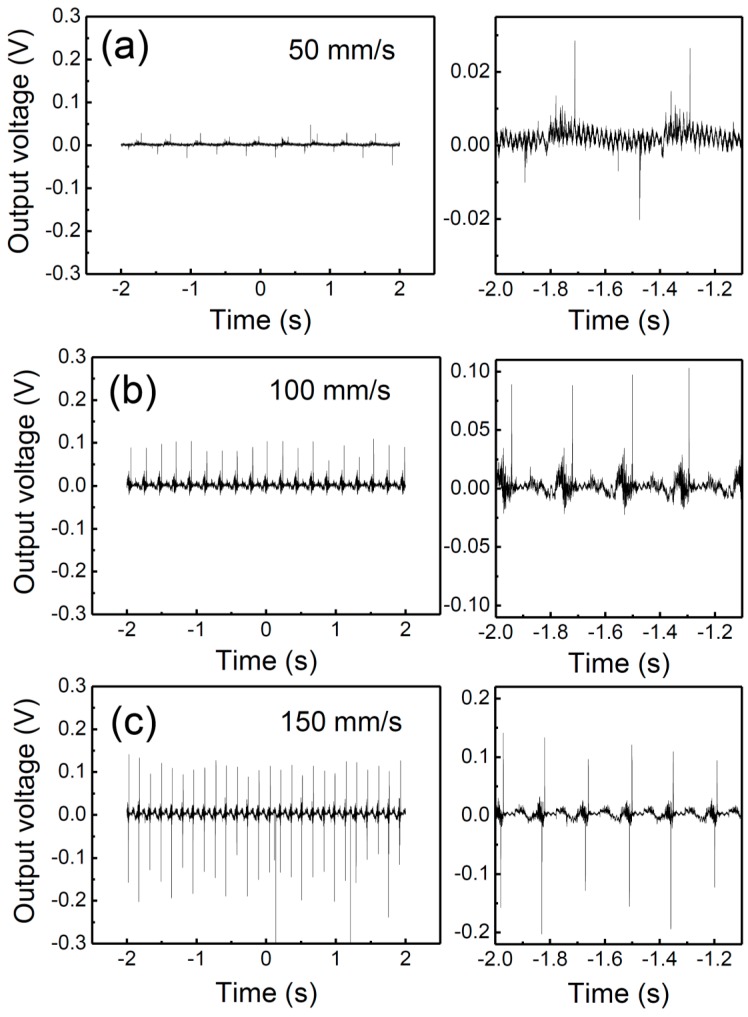
Variation in the NG properties according to the bending speed of (**a**) 50 mm/s; (**b**) 100 mm/s; and (**c**) 150 mm/s.

Figure 3 shows the variation in the NG properties according to the bending speed at the forward connection. When the bending speed increases to 50, 100, and 150 mm/s (units of moving distance per time), the output voltage enhances to 0.02, 0.09, and 0.15 V, respectively. When a bending speed of 150 mm/s is applied, the output voltage improvement is approximately 7–8 times when compared to the output voltage at 50 mm/s. As the bending speed increases to 50, 100, and 150 mm/s, the frequency also increases to 3, 5, and 7 Hz, respectively. This increase in frequency is closely related to the increase in the output voltage owing to their applied strain rate [17]. It can also be seen that the difference in the signals from the bending and releasing states decreases significantly with an increasing bending speed. This signal difference is owing to the different strain rates of the device during compression and the strain release [18]. Thus, the bending speed plays an important role in reducing the strain rate difference.

The main factors for bonding between CNT and polymer are mechanical interlocking (or friction), van der Waals interaction, and chemical bonding [19,20] Several bonds between them affect the stress transferring mechanism, facilitating the fabrication of reinforced polymer composites. In the case of PZTNP-CNT, the surface roughness is increased due to the presence of PZTNP on the CNT surface, which may increase the adhesion between polymer and CNT [21]. This adhesion prevents slipping of PZTNP-CNT in PTFE polymer and facilitates strain transfer between them. The high aspect ratio of the CNT also improves the ability to transfer the strain into the polymer, which is consistent with the case of cellulose with similar aspect ratios to CNT [22]. In addition, the friction between the CNT and the polymer increases when bending, and this friction plays an important role in transferring the strain into the PZTNP-CNT. Therefore, improvement of the PNG properties according to the bending-speed can be attributed to such bonding.

**Figure 4:**
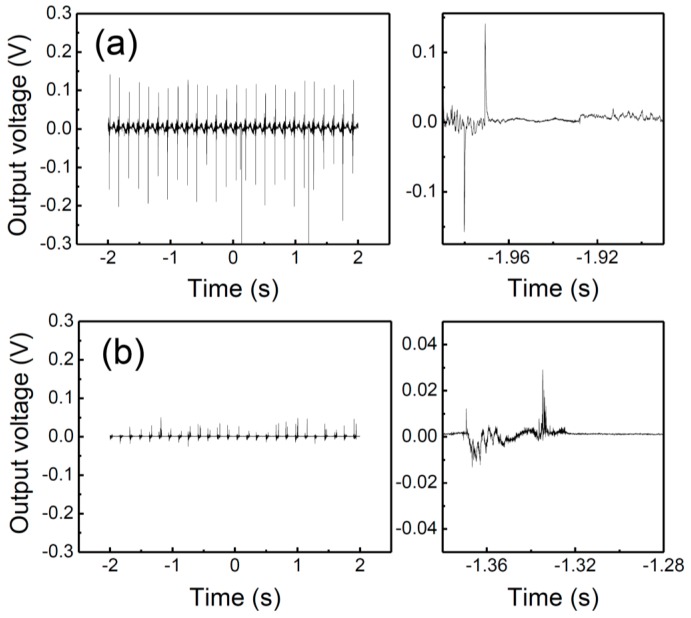
NG properties measured for the samples of (**a**) PZTNP-CNT and (**b**) mixed(PZTNP-CNT).

Figure 4 is a graph showing the NG properties measured for the PZTNP-CNT and mixed(PZTNP-CNT) samples of to identify the advantage of the direct growth of the PZTNPs on the CNTs. The mixed(PZTNP-CNT) sample was prepared by mixing PZTNPs crystallized by annealing and CNTs in the same ratio as in PZTNP-CNT. The developed voltage of mixed(PZTNP-CNT) was approximately five times smaller than that of PZTNP-CNT. In addition, the time difference between the bending and releasing was approximately four times slower than for PZTNP-CNT. The improvement in the power generation properties and reduction in the reaction time can be attributed to the direct growth of the PZTNPs on the CNTs. When a strain is applied, a charge carrier is formed in the piezoelectric PZTNP because of the piezoelectric effect, and it accumulates on both the electrodes. Here, the CNTs play an important role in accelerating the accumulation of charges on the electrode. The PZTNPs and CNTs being directly connected to each other, the amount of the charge carrier increases when the PZTNPs directly grow on the CNTs.

**Figure 5:**
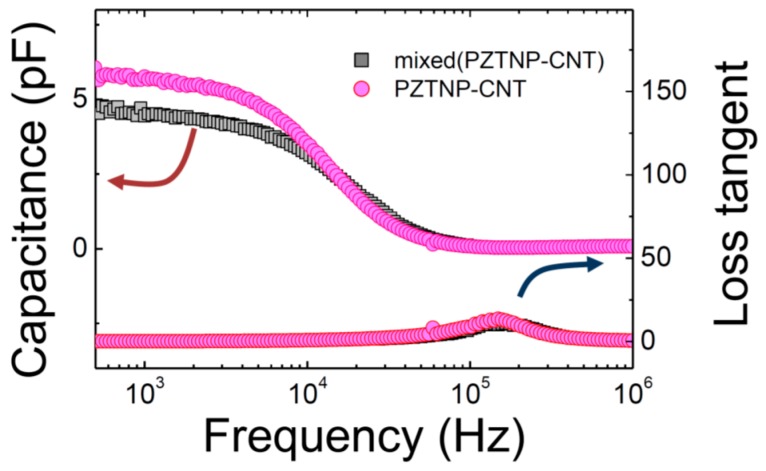
Frequency-dependence of the capacitance and dielectric loss of PZTNP-CNT and mixed(PZTNP-CNT).

Figure 5 shows the dielectric constant of the frequency dependent PZTNP-CNT composites. Admittance was measured by voltage and current as follows using dielectric constant measurement equipment [23].

(3) $$Y=\frac{I}{V}=\frac{R-i\left(X_{L}-X_{C}\right)}{R^{2}+{\left(X_{L}-X_{C}\right)}^{2}}$$

(4) $$X_{C}=\frac{1}{2\pi fC}$$

(5) $$X_{L}=2\pi fL$$

where *R* is resistance, *X*_C_ is capacitive reactance, and *X*_L_ is inductive reactance. There was no significant difference in the high frequency range, but the dielectric constant of PZTNP-CNT was slightly higher than that of mixed (PZTNP-CNT) in the lower frequency range. The dielectric constants of PZTNP-CNT and mixed (PZTNP-CNT) at 1 kHz were 4.5, 5.7 pico (10^−12^) Farad (pF), respectively.

This is probably due to the decrease in oxygen vacancy due to the interaction between CNT and PZT because they can fill with the oxygen in the surface of CNT during the annealing process. When the lattice oxygen of the CNT and the PZT unit cell reacts, oxygen vacancy, reduced Ti, and electrons are produced, which is similar to the Mars-van Krevelen mechanism [24]:

C + Ti^4+^ + O^2−^ → CO + V_O_ + Ti^3+^ + e^−^ (6)

where V_O_ is the oxygen vacancy and e^−^ is the charged electron.

During the heat treatment, oxygen vacancies can be filled by oxygen (carboxylic groups) on the surface of the CNT due to functionalization, resulting in a decrease in oxygen vacancies and consequently a high dielectric constant value. The loss tangent of PZTNP-CNT and mixed (PZTNP-CNT) was 0.1 and 0.13 at 1 kHz, respectively. The loss tangent of PZTNP-CNT was slightly lower than that of mixed(PZTNP-CNT), which is also believed to be due to a decrease in oxygen vacancy.

**Figure 6:**
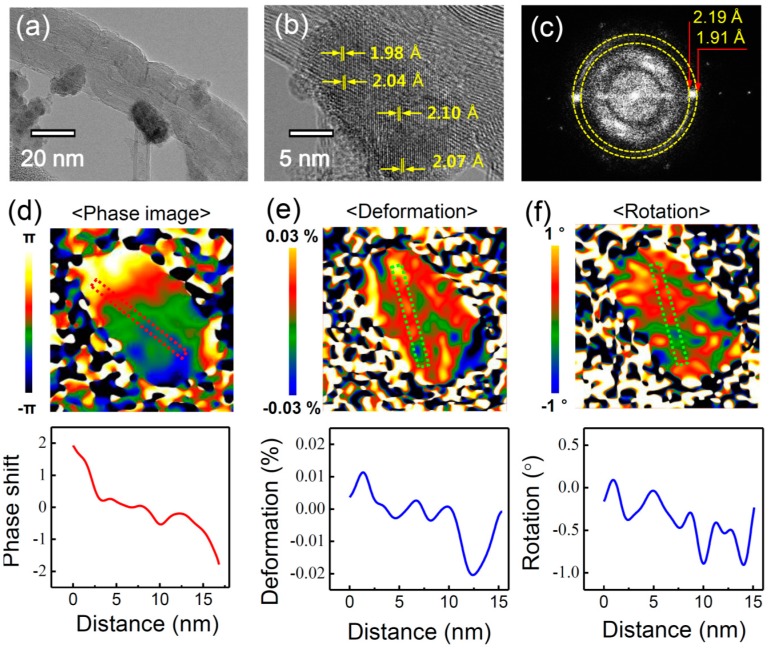
(**a**,**b**) FETEM images showing a PZTNP grown on a CNT surface; and the (**c**) FFT power spectrum; (**d**) phase; (**e**) deformation; and (**f**) rotation map of the (002) direction of PZTNP obtained from geometry phase analysis (GPA) analysis.

PZTNP-CNT was analyzed by TEM to determine the origin of their enhancement of the output voltage compared with mixed(PZTNP-CNT). Figure 6a shows the TEM images capturing the growth of a PZTNP on a CNT surface. The PZTNP is approximately 20 nm and shows a good growth on the CNT surface. Figure 6b displays an enlargement of the PZTNP image. The distance of the unit cell on the side with CNT is 1.98 Å, while the distance on the side without CNT is 2.07 Å; thus, the distance increases gradually in the growth direction of the PZTNP. Considering that the bulk value of perovskite (002) is 2.07 Å, it can be considered that the size is influenced by the unit cell of the PZTNP on the CNT, and it gradually becomes closer to the bulk value. Figure 6c presents the Fourier transform (FFT) spectrum for the PZTNP region. The value of Perovskite (002) was found to range from 1.91 Å to 2.19 Å, which is consistent with the lattice measurement in the TEM results. Figure 6d–f shows the phase, deformation, and rotation map, respectively, of the (002) direction of the PZTNP obtained from geometry phase analysis (GPA) analysis. Similar to the TEM results, near the CNT, the deformation and rotation of the lattice of the PZTNP decreases as it goes outward. This suggests that the unit cell is relaxed as the lattice of the PZTNP formed on the CNT grows. The structural change in the lattice indicates a strain gradient that suggests that the flexoelectric effect may affect the NG properties [25]. Therefore, the enhancement of the NG characteristic of a PZTNP grown directly on a CNT appears to be because this flexoelectric effect is generated in addition to the piezoelectric effect.

Their adsorption and migration energy are very important in the growth of metal oxide on CNT surface. Studies of the atomic migration and stability on graphene following the analysis of electronic structures using the first-principle calculation technique show that non-metallic elements such as PZT are mainly adsorbed between the nearest carbon atoms in the hexagonal structure of graphene [26]. When comparing the amount of adsorption energy for each element, the Pb atom (1.30 eV) is lower than the other ones (Zr: 3.42 eV, Ti: 3.27 eV, O: 4.79 eV) (1.42 Å), we suggest that Pb is first adsorbed to form a seed and PZT unit cell grows based on this. The tetragonal PZT grown on the hexagonal graphene has very different lattice mismatch due to its different structure and size [13]. This lattice mismatch causes a strain gradient in the NP.

We suggest that strain gradients inside the domain of oxide epitaxial nanostructures provide a highly interesting opportunity in future applications such as an energy storing fabric in the field of wearable devices. It has been reported that the strain gradient of epitaxial thin films is 0.01/(10 nm) = 10^6^ m^−1^, which is six or seven orders of magnitude larger than that (approximately 0.1 m^−1^) induced by the mechanical bending of bulk solids [27]. It has also been reported that the relaxation of strain can generate strain gradients of 10^7^ m^−1^ near misfit dislocations. According to the flexoelectric effect, this large strain gradient can induce an electric field of the order of 1−10 MV m^−1^, large enough to influence the physical properties of the films. In nanostructures such as nanoparticles, nanowires, and nanotubes, strain relaxation is very easy, causing an extraordinary large flexoelectric effect [13]. It is known that metal-oxide materials are hard materials as they are completely different from normal metals. Based on our TEM results, we suggest that such a strong softness of the metal oxide in nanostructures will be a powerful medium in strain control. In addition, our experiments support strong points regarding metal oxide ferroelectric nanostructures; (i) to strengthen the original dielectric polarization by flexoelectricity or to generate a new type of polarization in any insulating material, irrespective of its crystallographic symmetry; (ii) no requirement of the usual poling process because of the self-poling due to the strain gradient inside domain; (iii) the special role of CNT in our samples: a transportation of the stress through CNT depending on its conductivity. Therefore, we suggest that the flexoelectricity in ferroelectric nanomaterials can provide more functionalities, owing to the coexistence of flexoelectricity and ferroelectricity. Our next work is a piezoelectric/ferroelectric energy storing fabric. To weave with composite nanostructures, we are performing experiments by using the method of electro-spinning or wet-spinning.

## 3. Experimental Section

We conducted the pretreatment of CNTs through three steps. The as-grown CNTs were oxidized at 550 °C in air to remove the carbonaceous impurities. Subsequently, the oxidized CNTs were mixed in 6 M HCl solution with a ratio of 0.1 wt % and stirred, called the purification step. The HCl-treated CNTs were mixed in HNO_3_/H_2_SO_4_ solutions and stirred at 80 °C to functionalize them. The PZTNP-CNT was prepared by refluxing and filtering with a syringe filter of the mixed solution, including PZT and functionalized CNTs. A homogeneous PZT precursor solution was prepared via a modified 2-methoxyethanol-based sol-gel process. The functionalized CNTs were sonicated in a 0.3-M PZT precursor solution until a good suspension was obtained. The mixed solution was heated at 60 °C for 36 h and stirred via a magnetic bar in a two-necked round-bottom flask equipped with a water-cooled reflux condenser, called the reflux process, to fertilize the decorating PZTNPs on the surface of CNTs. The filtered powder was dried overnight at 80 °C. The powder was ground to a fine powder using a mortar and pestle, pyrolyzed at 400 °C in air, and annealed at 500‒700 °C in a nitrogen atmosphere. Flexible PZTNP-CNT films were fabricated by mixing the PZTNP-CNT powder with the PTFE as binders. Au/Cr electrodes were deposited on a Kapton film by a thermal evaporator. Prior to the attachment of the composite films to the electrode, poly(methyl methacrylate) (PMMA) was spin-coated on the Au/Cr/Kapton film. PMMA acted as an insulating layer to avoid electrical contact between the composite films and electrode. To enhance the piezoelectric effect of films, we applied DC 2.5 kV mm^−1^ across the electrodes at 150 °C for 48 h for electrical polarity (Stanford Research Systems, Inc. PS370, Sunnyvale, CA, USA). The output voltage was measured using an oscilloscope (Tektronix DPO 4054B, 500 MHz, Beaverton, OR, USA), applying a periodic bending movement via a cyclic bending machine (Radius bending tester, JIBT-610, Junil Tech, Daegu, South Korea).

## 4. Conclusions

In summary, we report the direct-growth of PZT on CNTs for piezoelectric nanogenerators. PZTNP-CNT shows detailed atomic scale-crystal growth of PZTNPs on the multi-walled CNTs that was confirmed by the FETEM analysis. The PZTNP-CNT are controlled the crystal orientation of the PZTNPs by adding a stirring process to the injection method. The PZT-atomically-interconnected CNTs caused an enhancement of the output voltage by an external mechanical force as compared with that of the simply-mixed PZT and CNTs. The nanogenerators repeatedly generate a voltage output of 0.19 V on bending at a bending speed of 150 mm/s that was higher than that of the simply-mixed PZT and MWCNTs. The output performance could be further optimized by increasing the internal resistance by applying a high poling voltage. Our results represent a momentous step toward application of nanogenerators in flexible electronics, portable devices, and mechanical sensors.

## Supplementary Materials

The following are available online at http://www.mdpi.com/2079-4991/7/10/308/s1, Figure S1: External resistance dependence of NG properties, Figure S2: NG properties using CNT films at forward and reverse connection, Table S1: Previous results of NG characteristics using PZT nanostructures.
