# Supplementary material for: Flexible Piezoelectric Generators by Using the Bending Motion Method of Direct-Grown-PZT Nanoparticles on Carbon Nanotubes
Source: Nanomaterials (Basel). 2017 Oct 7;7(10):308. doi: 10.3390/nano7100308 (PMC5666473; doi:10.3390/nano7100308)
Supplement: Supplementary file 1 [file nanomaterials-07-00308-s001.pdf]

Flexible Piezoelectric Generators by Using the Bending Motion Method of Direct-Grown-PZT Nanoparticles on Carbon Nanotubes

Jin Kyu Han [1,2], Do Hyun Jeon [1], Sam Yeon Cho [1], Sin Wook Kang [1], Jongsun Lim [2] and Sang Don Bu [1,*]

[1] Department of Physics and Research Institute of Physics and Chemistry, Chonbuk National University, Jeonju 54896, Korea; jkhan@jbnu.ac.kr (J.K.H.); dhjeon@jbnu.ac.kr (D.H.J.); syc@jbnu.ac.kr (S.Y.C.); swkang@jbnu.ac.kr (S.W.K.)
[2] Thin Film Materials Research Center, Korea Research Institute of Chemical Technology (KRICT), Daejeon 34114, Korea; jslim@krict.re.kr
* Correspondence: sbu@jbnu.ac.kr; Tel.: +82-63-270-4264

Table S1. Previous results of NG characteristics using PZT nanostructures

Materials	Bending	Tapping	Ref
PZT nanowire		1.63 V	[1]
PZT particle		1.2 V	[2]
PZT nanowire		0.9 V	[3]
PZTNP-CNT-PDMS composite		8.6 V	[4]
PZTNP-CNT-PTFE composite	0.19 V		This work

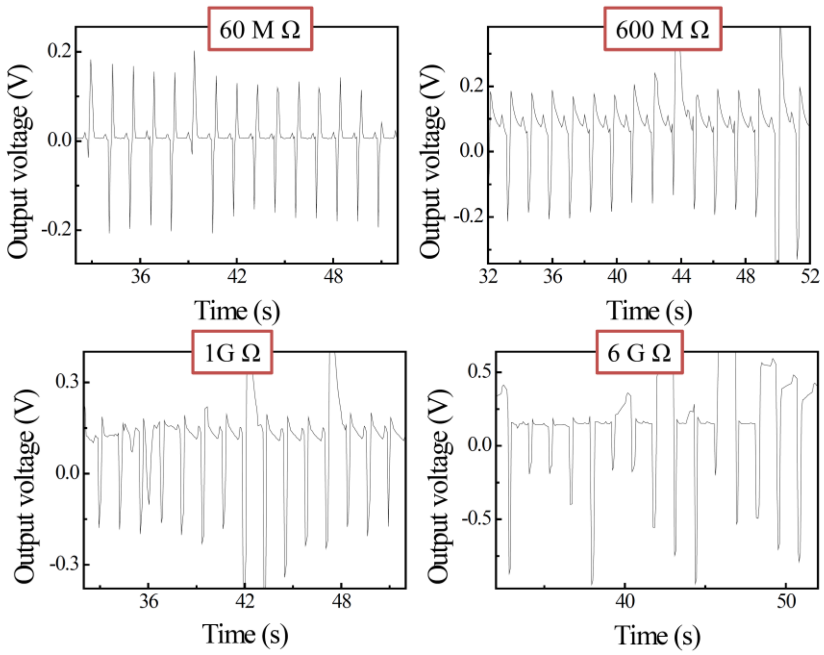

Figure S1. External resistance dependence of NG properties

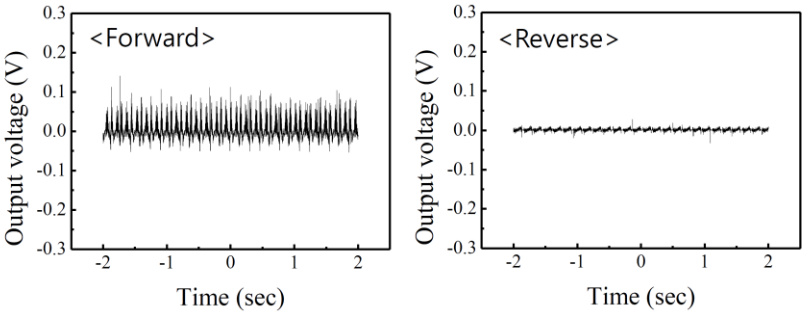

Figure S2. NG properties using CNT films at forward and reverse connection.

References

1. Chen, X.; Xu, S.; Yao, N.; Shi, Y. 1.6 V Nanogenerator for Mechanical Energy Harvesting Using PZT Nanofibers. *Nano letters* **2010**, *10*, 2133-2137.
2. Zhang, Y.; Zhang, Y.; Xue, X.; Cui, C.; He, B.; Nie, Y.; Deng, P.; Wang, Z.L. PVDF-PZT nanocomposite film based self-charging power cell. *Nanotechnology* **2014**, *25*, 105401.
3. Jung, W.-S.; Lee, W.-H.; Ju, B.-K.; Yoon, S.-J.; Kang, C.-Y. Enhanced output power of PZT nanogenerator by controlling surface morphology of electrode. *Journal of nanoscience and nanotechnology* **2015**, *15*, 8907-8911.
4. Han, J.K.; Do Hyun Jeon, S.Y.C.; Kang, S.W.; Yang, S.A.; Bu, S.D.; Myung, S.; Lim, J.; Choi, M.; Lee, M.; Lee, M.K. Nanogenerators consisting of direct-grown piezoelectrics on multi-walled carbon nanotubes using flexoelectric effects. *Scientific reports* **2016**, *6*, 29562.
